# 1-Isopropyl-4,7-dimethyl-2,8-dinitro­naphthalene

**DOI:** 10.1107/S1600536812021514

**Published:** 2012-05-26

**Authors:** Mouna Chakkar, Najia Oughris, Ahmed Benharref, Jean-Claude Daran, Moha Berraho

**Affiliations:** a Laboratoire de Chimie des Substances Naturelles, URAC16, Faculté des Sciences Semlalia, BP 2390 Bd My Abdellah, 40000 Marrakech, Morocco; b Laboratoire de Chimie de Coordination, 205 route de Narbonne, 31077 Toulouse Cedex 04, France

## Abstract

The title compound, C_15_H_16_N_2_O_4_, was synthesized from a mixture of α-himachalene (2-methyl­ene-6,6,9-trimethyl­bicyclo­[5.4.O^1,7^]undec-8-ene) and β-himachalene (2,6,6,9-tetra­methyl­bicyclo­[5.4.0^1,7^]undeca-1,8-diene) which were isolated from an oil of the Atlas cedar (*Cedrus atlantica*). The asymmetric unit contains two independent mol­ecules. In each of the two mol­ecules, two O atoms of one nitro group are disordered over two sets of sites with site-occupancy factors of 0.636 (5):0.364 (5) and 0.832 (5):0.168 (5). The crystal structure features weak C—H⋯O hydrogen bonds.

## Related literature

For the isolation of α-himachalene and β-himachalene, see: Joseph & Dev (1968 ▶); Plattier & Teisseire (1974 ▶); Daunis *et al.* (1981 ▶). For the reactivity of this sesquiterpene, see: Lassaba *et al.* (1998 ▶); Chekroun *et al.* (2000 ▶); El Jamili *et al.* (2002 ▶); Sbai *et al.* (2002 ▶); Dakir *et al.* (2004 ▶). For its biological activity, see: Daoubi *et al.* (2004 ▶).
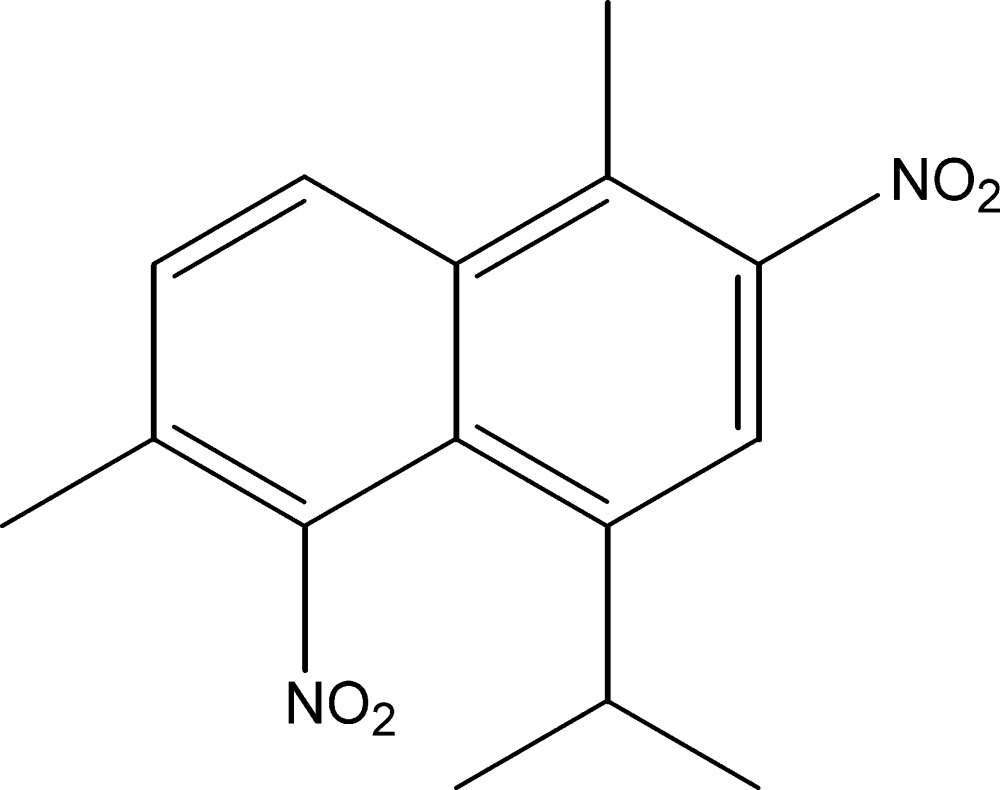


## Experimental

### 

#### Crystal data

C_15_H_16_N_2_O_4_
*M* *_r_* = 288.30
Triclinic, 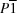

*a* = 11.7784 (7) Å
*b* = 11.9072 (9) Å
*c* = 12.4494 (10) Å
α = 107.928 (7)°
β = 112.834 (7)°
γ = 104.536 (6)°
*V* = 1387.6 (2) Å^3^
*Z* = 4
Mo *K*α radiation
μ = 0.10 mm^−1^
*T* = 180 K
0.49 × 0.22 × 0.14 mm

#### Data collection

Agilent Xcalibur Sapphire1 (long-nozzle) diffractometer
25504 measured reflections
4881 independent reflections
4084 reflections with *I* > 2σ(*I*)
*R*_int_ = 0.048

#### Refinement

*R*[*F* ^2^ > 2σ(*F* ^2^)] = 0.056
*wR*(*F* ^2^) = 0.152
*S* = 1.08
4881 reflections
425 parameters
H-atom parameters constrained
Δρ_max_ = 0.19 e Å^−3^
Δρ_min_ = −0.26 e Å^−3^

### 

Data collection: *CrysAlis PRO* (Agilent, 2010 ▶); cell refinement: *CrysAlis PRO*; data reduction: *CrysAlis PRO*; program(s) used to solve structure: *SHELXS97* (Sheldrick, 2008 ▶); program(s) used to refine structure: *SHELXL97* (Sheldrick, 2008 ▶); molecular graphics: *ORTEP-3 for Windows* (Farrugia, 1997 ▶) and *PLATON* (Spek, 2009 ▶); software used to prepare material for publication: *WinGX* (Farrugia, 1999 ▶).

## Supplementary Material

Crystal structure: contains datablock(s) I, global. DOI: 10.1107/S1600536812021514/bt5912sup1.cif

Structure factors: contains datablock(s) I. DOI: 10.1107/S1600536812021514/bt5912Isup2.hkl

Supplementary material file. DOI: 10.1107/S1600536812021514/bt5912Isup3.cml

Additional supplementary materials:  crystallographic information; 3D view; checkCIF report

## Figures and Tables

**Table 1:** Hydrogen-bond geometry (Å, °)

*D*—H⋯*A*	*D*—H	H⋯*A*	*D*⋯*A*	*D*—H⋯*A*
C11*B*—H11*C*⋯O82^i^	0.98	2.42	3.243 (6)	142
C11*A*—H11*F*⋯O84*A*^ii^	0.98	2.43	3.240 (5)	139

Symmetry codes: (i) 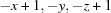; (ii) 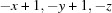.

**Fig. 1.**
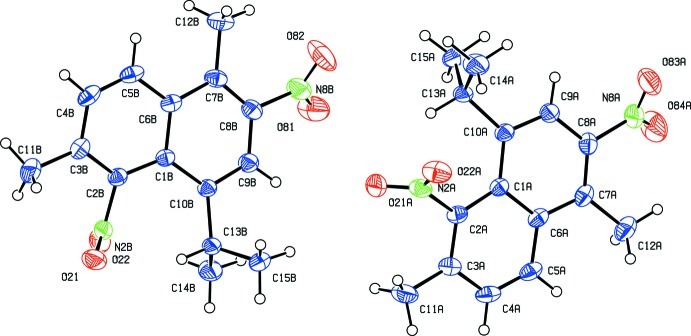
: Molecular structure of the title compound with the atom-labelling scheme. Displacement ellipsoids are drawn at the 30% probability level. H atoms are represented as small spheres of arbitrary radii.

**Fig. 2.**
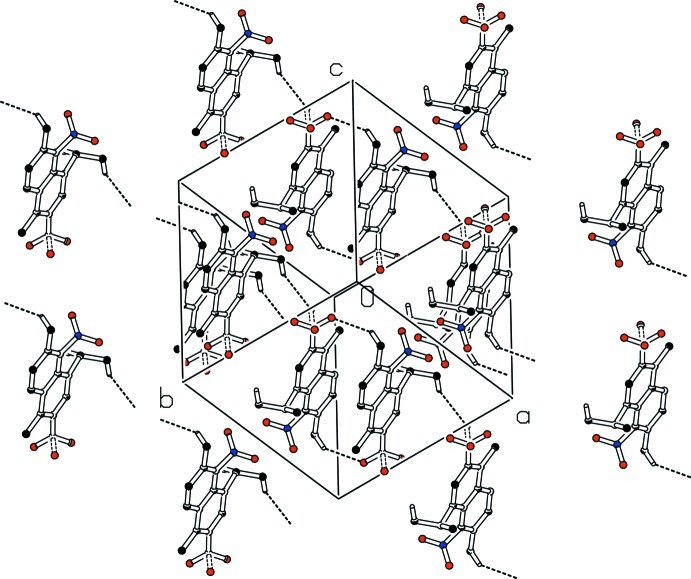
: Partial packing view showing the C—H···O interactions (dashed lines) and the formation of a chain parallel to the a axis. H atoms not involved in hydrogen bonding have been omitted for clarity.

## supplementary crystallographic information

### Comment

The bicyclic sesquiterpenes α- and β-himachalene are the main constituents of the essential oil of the Atlas cedar (Cedrus atlantica) (Joseph & Dev, 1968; Plattier & Teisseire, 1974). The reactivity of these sesquiterpenes and its derivatives has been studied extensively by our team in order to prepare new products having biological proprieties (Lassaba *et al.*, 1998; Chekroun *et al.*, 2000; El Jamili *et al.*, 2002; Sbai *et al.*, 2002; Dakir *et al.*, 2004). Indeed, these compounds were tested, using the food poisoning technique, for their potential antifungal activity against the phytopathogen Botrytis cinerea (Daoubi *et al.*, 2004). The catalytic dehydrogenation of the mixture of α- and β-himachalene by 5% of palladium on carbon(10%) gives, with good yield, the aryl-himachalene (Daunis *et al.*, 1981). Treatement of the latter by a mixture of nitric acid and sulfuric acid, gives the title compound with a yield of 20%. The structure of this new product was confirmed by its crystal structure. The molecular structure of (I) is shown in Fig. 1. The asymmetric unit contains two molecules of 1-isopropyl-4,7-dimethyl-2,8-dinitro-naphthalene. The naphthalene ring systems are approximately planar with r.s.d.deviations of 0.087 (2) and 0.090 (2) A°. The bond lengths and angles are within normal ranges in both molecules. In the crystal structure, the two molecules are not parallel but have a dihedral angle of 1.54 (7)°. The crystal structure is stabilized by intermolecular C–H···O hydrogen bonds, which link the molecules into chains parallel to the *c* axis (Fig. 2, Table 1).

### Experimental

In a reactor of 250 ml equipped with a magnetic stirrer and a dropping funnel, we introduct 60 ml of dichloromethane, 3 ml of nitric acid and 5 ml of concentrated sulfuric acid. After cooling, added dropwise through the dropping funnel 6 g (30 mmol) of aryl-himachalene dissolved in 30 ml of dichloromethane. The reaction mixture was stirred for 4 h, then added 50 ml of water ice and extracted with dichloromethane. The organic layers were combined, washed five times with 4O ml with water and dried over sodium sulfate and then concentrated under vacuum. The residue was subjected to chromatography on a column of silica gel with hexane-ethyl acetate (98/2) as eluent, to obtain 1.7 g (6 mmol) of the title compound which was recrystallized in ethyl acetate.

### Refinement

All H atoms were fixed geometrically and treated as riding with C—H = 0.96 Å (methyl),0.97 Å (methylene), 0.98 Å (methine) with *U*_iso_(H) = 1.2Ueq(methylene, methine) or *U*_iso_(H) = 1.5Ueq(methyl). In each of the two molecules, two O atoms of one nitro group are disordered over two positions with site occupancy factors of 0.636 (5)/0.364 (5) for the first molecule, and 0.832 (5)/0.168 (5) for the second molecule.

### Crystal data

**Table:**

C_15_H_16_N_2_O_4_	*Z* = 4
*M**_r_* = 288.30	*F*(000) = 608
Triclinic, *P*1	*D*_x_ = 1.380 Mg m^−^^3^
Hall symbol: -P 1	Mo *K*α radiation, λ = 0.71073 Å
*a* = 11.7784 (7) Å	Cell parameters from 13404 reflections
*b* = 11.9072 (9) Å	θ = 3.1–28.4°
*c* = 12.4494 (10) Å	µ = 0.10 mm^−^^1^
α = 107.928 (7)°	*T* = 180 K
β = 112.834 (7)°	Box, orange
γ = 104.536 (6)°	0.49 × 0.22 × 0.14 mm
*V* = 1387.6 (2) Å^3^	

### Data collection

**Table:**

Agilent Xcalibur Sapphire1 (long-nozzle) diffractometer	4084 reflections with *I* > 2σ(*I*)
Radiation source: fine-focus sealed tube	*R*_int_ = 0.048
Graphite monochromator	θ_max_ = 25.0°, θ_min_ = 3.1°
Detector resolution: 8.2632 pixels mm^-1^	*h* = −14→14
ω scans	*k* = −14→14
25504 measured reflections	*l* = −14→14
4881 independent reflections	

### Refinement

**Table:**

Refinement on *F*^2^	Primary atom site location: structure-invariant direct methods
Least-squares matrix: full	Secondary atom site location: difference Fourier map
*R*[*F*^2^ > 2σ(*F*^2^)] = 0.056	Hydrogen site location: inferred from neighbouring sites
*wR*(*F*^2^) = 0.152	H-atom parameters constrained
*S* = 1.08	*w* = 1/[σ^2^(*F*_o_^2^) + (0.0578*P*)^2^ + 0.9464*P*] where *P* = (*F*_o_^2^ + 2*F*_c_^2^)/3
4881 reflections	(Δ/σ)_max_ < 0.001
425 parameters	Δρ_max_ = 0.19 e Å^−^^3^
0 restraints	Δρ_min_ = −0.26 e Å^−^^3^

### Special details

**Table:**

Geometry. All s.u.'s (except the s.u. in the dihedral angle between two l.s. planes) are estimated using the full covariance matrix. The cell s.u.'s are taken into account individually in the estimation of s.u.'s in distances, angles and torsion angles; correlations between s.u.'s in cell parameters are only used when they are defined by crystal symmetry. An approximate (isotropic) treatment of cell s.u.'s is used for estimating s.u.'s involving l.s. planes.
Refinement. Refinement of *F*^2^ against ALL reflections. The weighted *R*-factor *wR* and goodness of fit *S* are based on *F*^2^, conventional *R*-factors *R* are based on *F*, with *F* set to zero for negative *F*^2^. The threshold expression of *F*^2^ > 2σ(*F*^2^) is used only for calculating *R*-factors(gt) *etc*. and is not relevant to the choice of reflections for refinement. *R*-factors based on *F*^2^ are statistically about twice as large as those based on *F*, and *R*- factors based on ALL data will be even larger.

### Fractional atomic coordinates and isotropic or equivalent isotropic displacement parameters (Å^2^)

**Table:**

	*x*	*y*	*z*	*U*_iso_*/*U*_eq_	Occ. (<1)
N2B	0.12177 (19)	−0.0333 (2)	0.42969 (19)	0.0400 (5)	
O21	0.04646 (17)	−0.14879 (18)	0.35187 (18)	0.0557 (5)	
O22	0.08307 (17)	0.05310 (19)	0.45226 (18)	0.0513 (5)	
C1B	0.3602 (2)	0.10012 (19)	0.49485 (19)	0.0280 (4)	
C2B	0.2692 (2)	0.0062 (2)	0.5059 (2)	0.0312 (5)	
C3B	0.3074 (2)	−0.0430 (2)	0.5940 (2)	0.0374 (5)	
C4B	0.4479 (2)	0.0090 (2)	0.6837 (2)	0.0412 (5)	
H4	0.4787	−0.0243	0.7443	0.049*	
C5B	0.5394 (2)	0.1052 (2)	0.6852 (2)	0.0386 (5)	
H5	0.6324	0.1410	0.7503	0.046*	
C6B	0.5015 (2)	0.1544 (2)	0.59319 (19)	0.0312 (5)	
C7B	0.6027 (2)	0.2564 (2)	0.5992 (2)	0.0356 (5)	
C8B	0.5566 (2)	0.2971 (2)	0.5057 (2)	0.0380 (5)	
N8B	0.6494 (2)	0.4042 (2)	0.5031 (2)	0.0534 (6)	
O81	0.6072 (3)	0.4850 (3)	0.4795 (4)	0.0800 (15)	0.636 (5)
O82	0.7589 (4)	0.4098 (4)	0.5207 (5)	0.0887 (16)	0.636 (5)
O81B	0.6349 (6)	0.3925 (7)	0.4012 (6)	0.080 (3)	0.364 (5)
O82B	0.7403 (6)	0.4984 (6)	0.6108 (6)	0.077 (2)	0.364 (5)
C9B	0.4217 (2)	0.2397 (2)	0.4043 (2)	0.0371 (5)	
H9	0.3981	0.2712	0.3410	0.044*	
C10B	0.3226 (2)	0.1397 (2)	0.3930 (2)	0.0305 (4)	
C11B	0.2082 (3)	−0.1448 (3)	0.6004 (3)	0.0533 (7)	
H11A	0.1297	−0.1252	0.5902	0.080*	
H11B	0.2525	−0.1450	0.6852	0.080*	
H11C	0.1777	−0.2306	0.5301	0.080*	
C12B	0.7496 (2)	0.3132 (3)	0.7049 (2)	0.0516 (6)	
H12A	0.7877	0.2502	0.6865	0.077*	
H12B	0.7553	0.3330	0.7898	0.077*	
H12C	0.8011	0.3934	0.7071	0.077*	
C13B	0.1839 (2)	0.0751 (2)	0.2675 (2)	0.0363 (5)	
H13	0.1263	−0.0044	0.2650	0.044*	
C14B	0.1119 (2)	0.1656 (3)	0.2664 (3)	0.0521 (7)	
H14A	0.1629	0.2413	0.2620	0.078*	
H14B	0.1068	0.1948	0.3464	0.078*	
H14C	0.0198	0.1187	0.1900	0.078*	
C15B	0.1981 (2)	0.0324 (3)	0.1465 (2)	0.0468 (6)	
H15A	0.2485	0.1094	0.1434	0.070*	
H15B	0.1076	−0.0167	0.0679	0.070*	
H15C	0.2473	−0.0229	0.1504	0.070*	
N2A	0.54123 (18)	0.18844 (18)	0.0592 (2)	0.0411 (5)	
O21A	0.50204 (18)	0.18713 (19)	0.13648 (19)	0.0555 (5)	
O22A	0.60540 (17)	0.12814 (16)	0.03450 (18)	0.0531 (5)	
C1A	0.6104 (2)	0.36905 (19)	0.00222 (19)	0.0286 (4)	
C2A	0.5054 (2)	0.2631 (2)	−0.0138 (2)	0.0322 (5)	
C3A	0.3692 (2)	0.2123 (2)	−0.1043 (2)	0.0370 (5)	
C4A	0.3327 (2)	0.2679 (2)	−0.1899 (2)	0.0411 (6)	
H4A	0.2395	0.2379	−0.2517	0.049*	
C5A	0.4280 (2)	0.3635 (2)	−0.1861 (2)	0.0385 (5)	
H5A	0.3999	0.3957	−0.2482	0.046*	
C6A	0.5681 (2)	0.4170 (2)	−0.09219 (19)	0.0321 (5)	
C7A	0.6641 (2)	0.5173 (2)	−0.0930 (2)	0.0363 (5)	
C8A	0.7973 (2)	0.5639 (2)	0.0014 (2)	0.0378 (5)	
N8A	0.9075 (2)	0.6642 (3)	0.0088 (3)	0.0509 (6)	
O84A	0.8930 (3)	0.7589 (3)	−0.0031 (3)	0.0754 (10)	0.832 (5)
O83A	1.0134 (3)	0.6522 (3)	0.0337 (3)	0.0739 (10)	0.832 (5)
O83	0.8868 (16)	0.6378 (15)	−0.1107 (14)	0.092 (6)	0.168 (5)
O84	0.985 (2)	0.7387 (19)	0.0982 (18)	0.100 (7)	0.168 (5)
C9A	0.8398 (2)	0.5236 (2)	0.0982 (2)	0.0361 (5)	
H9A	0.9337	0.5613	0.1613	0.043*	
C10A	0.7506 (2)	0.4317 (2)	0.1052 (2)	0.0315 (5)	
C11A	0.2617 (2)	0.1001 (3)	−0.1173 (3)	0.0541 (7)	
H11D	0.2949	0.0335	−0.1105	0.081*	
H11E	0.1791	0.0626	−0.2025	0.081*	
H11F	0.2414	0.1314	−0.0475	0.081*	
C12A	0.6183 (3)	0.5633 (3)	−0.1961 (2)	0.0510 (6)	
H12D	0.6971	0.6114	−0.1988	0.077*	
H12E	0.5751	0.6203	−0.1747	0.077*	
H12F	0.5531	0.4879	−0.2816	0.077*	
C13A	0.8082 (2)	0.4112 (2)	0.2271 (2)	0.0380 (5)	
H13A	0.7303	0.3530	0.2270	0.046*	
C14A	0.9031 (3)	0.3446 (3)	0.2262 (3)	0.0558 (7)	
H14D	0.8544	0.2618	0.1464	0.084*	
H14E	0.9340	0.3283	0.3029	0.084*	
H14F	0.9817	0.4010	0.2289	0.084*	
C15A	0.8806 (3)	0.5412 (3)	0.3512 (2)	0.0520 (7)	
H15D	0.9080	0.5254	0.4282	0.078*	
H15E	0.8187	0.5836	0.3479	0.078*	
H15F	0.9612	0.5978	0.3567	0.078*	

### Atomic displacement parameters (Å^2^)

**Table:**

	*U*^11^	*U*^22^	*U*^33^	*U*^12^	*U*^13^	*U*^23^
N2B	0.0325 (10)	0.0534 (13)	0.0430 (11)	0.0181 (10)	0.0194 (9)	0.0319 (10)
O21	0.0365 (9)	0.0521 (11)	0.0577 (11)	0.0039 (8)	0.0110 (8)	0.0297 (9)
O22	0.0449 (10)	0.0755 (13)	0.0656 (12)	0.0388 (10)	0.0364 (9)	0.0475 (10)
C1B	0.0280 (10)	0.0284 (10)	0.0274 (10)	0.0150 (8)	0.0124 (8)	0.0117 (8)
C2B	0.0309 (11)	0.0349 (11)	0.0294 (10)	0.0173 (9)	0.0141 (9)	0.0152 (9)
C3B	0.0449 (13)	0.0403 (12)	0.0319 (11)	0.0215 (10)	0.0201 (10)	0.0184 (10)
C4B	0.0510 (14)	0.0487 (14)	0.0305 (11)	0.0310 (12)	0.0169 (10)	0.0223 (10)
C5B	0.0370 (12)	0.0441 (13)	0.0281 (11)	0.0244 (11)	0.0087 (9)	0.0130 (10)
C6B	0.0317 (11)	0.0320 (11)	0.0262 (10)	0.0178 (9)	0.0118 (9)	0.0091 (9)
C7B	0.0284 (11)	0.0322 (11)	0.0333 (11)	0.0124 (9)	0.0115 (9)	0.0058 (9)
C8B	0.0330 (11)	0.0310 (11)	0.0413 (12)	0.0077 (9)	0.0171 (10)	0.0133 (10)
N8B	0.0397 (12)	0.0451 (13)	0.0546 (15)	0.0039 (10)	0.0154 (11)	0.0207 (12)
O81	0.058 (2)	0.055 (2)	0.100 (3)	0.0064 (16)	0.0132 (19)	0.052 (2)
O82	0.049 (2)	0.093 (3)	0.143 (4)	0.0239 (19)	0.056 (2)	0.073 (3)
O81B	0.065 (4)	0.080 (5)	0.050 (4)	−0.017 (3)	0.023 (3)	0.025 (3)
O82B	0.057 (4)	0.051 (4)	0.074 (4)	−0.012 (3)	0.032 (3)	0.003 (3)
C9B	0.0333 (11)	0.0369 (12)	0.0384 (12)	0.0131 (10)	0.0141 (10)	0.0207 (10)
C10B	0.0282 (10)	0.0320 (11)	0.0321 (11)	0.0146 (9)	0.0130 (9)	0.0169 (9)
C11B	0.0593 (16)	0.0582 (16)	0.0484 (15)	0.0211 (13)	0.0264 (13)	0.0353 (13)
C12B	0.0312 (12)	0.0538 (15)	0.0452 (14)	0.0127 (11)	0.0082 (11)	0.0129 (12)
C13B	0.0270 (10)	0.0426 (12)	0.0381 (12)	0.0117 (9)	0.0108 (9)	0.0266 (10)
C14B	0.0374 (13)	0.0678 (17)	0.0646 (17)	0.0292 (12)	0.0213 (12)	0.0460 (15)
C15B	0.0386 (13)	0.0566 (15)	0.0350 (12)	0.0129 (11)	0.0106 (10)	0.0251 (11)
N2A	0.0302 (10)	0.0333 (10)	0.0468 (11)	0.0075 (8)	0.0087 (9)	0.0221 (9)
O21A	0.0447 (10)	0.0669 (12)	0.0605 (11)	0.0169 (9)	0.0240 (9)	0.0441 (10)
O22A	0.0439 (10)	0.0380 (9)	0.0684 (12)	0.0221 (8)	0.0159 (9)	0.0260 (9)
C1A	0.0313 (10)	0.0306 (10)	0.0278 (10)	0.0183 (9)	0.0145 (9)	0.0142 (9)
C2A	0.0311 (11)	0.0314 (11)	0.0308 (11)	0.0152 (9)	0.0113 (9)	0.0141 (9)
C3A	0.0300 (11)	0.0364 (12)	0.0349 (11)	0.0141 (9)	0.0116 (9)	0.0111 (10)
C4A	0.0318 (11)	0.0479 (14)	0.0307 (11)	0.0221 (11)	0.0068 (9)	0.0107 (10)
C5A	0.0407 (12)	0.0484 (14)	0.0292 (11)	0.0282 (11)	0.0133 (10)	0.0190 (10)
C6A	0.0377 (11)	0.0376 (12)	0.0269 (10)	0.0247 (10)	0.0160 (9)	0.0147 (9)
C7A	0.0483 (13)	0.0411 (12)	0.0332 (11)	0.0271 (11)	0.0239 (10)	0.0218 (10)
C8A	0.0418 (12)	0.0405 (12)	0.0423 (12)	0.0196 (10)	0.0258 (11)	0.0238 (10)
N8A	0.0493 (14)	0.0586 (15)	0.0548 (15)	0.0195 (12)	0.0284 (12)	0.0370 (13)
O84A	0.0739 (18)	0.0607 (17)	0.111 (2)	0.0272 (14)	0.0484 (17)	0.0601 (17)
O83A	0.0475 (15)	0.104 (2)	0.102 (2)	0.0338 (15)	0.0435 (15)	0.074 (2)
O83	0.110 (12)	0.088 (11)	0.084 (10)	0.010 (9)	0.072 (10)	0.040 (9)
O84	0.086 (12)	0.092 (13)	0.079 (12)	−0.013 (11)	0.032 (10)	0.041 (10)
C9A	0.0326 (11)	0.0399 (12)	0.0389 (12)	0.0162 (10)	0.0163 (10)	0.0229 (10)
C10A	0.0302 (10)	0.0344 (11)	0.0325 (11)	0.0173 (9)	0.0137 (9)	0.0184 (9)
C11A	0.0320 (12)	0.0507 (15)	0.0519 (15)	0.0063 (11)	0.0082 (11)	0.0174 (13)
C12A	0.0633 (16)	0.0617 (16)	0.0449 (14)	0.0343 (14)	0.0286 (13)	0.0355 (13)
C13A	0.0258 (10)	0.0446 (13)	0.0404 (12)	0.0111 (9)	0.0093 (9)	0.0283 (11)
C14A	0.0409 (13)	0.0651 (17)	0.0715 (18)	0.0295 (13)	0.0199 (13)	0.0482 (15)
C15A	0.0418 (13)	0.0590 (16)	0.0378 (13)	0.0104 (12)	0.0080 (11)	0.0264 (12)

### Geometric parameters (Å, º)

**Table:**

N2B—O22	1.226 (3)	N2A—O21A	1.220 (3)
N2B—O21	1.231 (3)	N2A—O22A	1.224 (3)
N2B—C2B	1.474 (3)	N2A—C2A	1.477 (3)
C1B—C2B	1.423 (3)	C1A—C2A	1.425 (3)
C1B—C10B	1.436 (3)	C1A—C6A	1.435 (3)
C1B—C6B	1.442 (3)	C1A—C10A	1.441 (3)
C2B—C3B	1.376 (3)	C2A—C3A	1.378 (3)
C3B—C4B	1.413 (3)	C3A—C4A	1.408 (3)
C3B—C11B	1.505 (3)	C3A—C11A	1.507 (3)
C4B—C5B	1.350 (3)	C4A—C5A	1.357 (3)
C4B—H4	0.9500	C4A—H4A	0.9500
C5B—C6B	1.415 (3)	C5A—C6A	1.419 (3)
C5B—H5	0.9500	C5A—H5A	0.9500
C6B—C7B	1.432 (3)	C6A—C7A	1.429 (3)
C7B—C8B	1.368 (3)	C7A—C8A	1.371 (3)
C7B—C12B	1.510 (3)	C7A—C12A	1.510 (3)
C8B—C9B	1.398 (3)	C8A—C9A	1.394 (3)
C8B—N8B	1.475 (3)	C8A—N8A	1.476 (3)
N8B—O81B	1.170 (6)	N8A—O84	1.028 (18)
N8B—O82	1.201 (4)	N8A—O83A	1.220 (3)
N8B—O82B	1.248 (6)	N8A—O84A	1.226 (3)
N8B—O81	1.249 (4)	N8A—O83	1.328 (13)
C9B—C10B	1.369 (3)	C9A—C10A	1.363 (3)
C9B—H9	0.9500	C9A—H9A	0.9500
C10B—C13B	1.533 (3)	C10A—C13A	1.529 (3)
C11B—H11A	0.9800	C11A—H11D	0.9800
C11B—H11B	0.9800	C11A—H11E	0.9800
C11B—H11C	0.9800	C11A—H11F	0.9800
C12B—H12A	0.9800	C12A—H12D	0.9800
C12B—H12B	0.9800	C12A—H12E	0.9800
C12B—H12C	0.9800	C12A—H12F	0.9800
C13B—C15B	1.524 (3)	C13A—C14A	1.525 (3)
C13B—C14B	1.530 (3)	C13A—C15A	1.534 (3)
C13B—H13	1.0000	C13A—H13A	1.0000
C14B—H14A	0.9800	C14A—H14D	0.9800
C14B—H14B	0.9800	C14A—H14E	0.9800
C14B—H14C	0.9800	C14A—H14F	0.9800
C15B—H15A	0.9800	C15A—H15D	0.9800
C15B—H15B	0.9800	C15A—H15E	0.9800
C15B—H15C	0.9800	C15A—H15F	0.9800
			
O22—N2B—O21	124.71 (19)	O21A—N2A—O22A	124.2 (2)
O22—N2B—C2B	116.18 (19)	O21A—N2A—C2A	118.68 (19)
O21—N2B—C2B	119.07 (19)	O22A—N2A—C2A	117.1 (2)
C2B—C1B—C10B	125.38 (18)	C2A—C1A—C6A	115.35 (18)
C2B—C1B—C6B	115.49 (18)	C2A—C1A—C10A	125.69 (18)
C10B—C1B—C6B	119.10 (18)	C6A—C1A—C10A	118.94 (18)
C3B—C2B—C1B	125.10 (19)	C3A—C2A—C1A	125.33 (19)
C3B—C2B—N2B	114.96 (19)	C3A—C2A—N2A	114.58 (19)
C1B—C2B—N2B	119.57 (17)	C1A—C2A—N2A	119.71 (17)
C2B—C3B—C4B	116.7 (2)	C2A—C3A—C4A	116.7 (2)
C2B—C3B—C11B	123.6 (2)	C2A—C3A—C11A	123.4 (2)
C4B—C3B—C11B	119.7 (2)	C4A—C3A—C11A	119.9 (2)
C5B—C4B—C3B	121.4 (2)	C5A—C4A—C3A	121.3 (2)
C5B—C4B—H4	119.3	C5A—C4A—H4A	119.3
C3B—C4B—H4	119.3	C3A—C4A—H4A	119.3
C4B—C5B—C6B	122.3 (2)	C4A—C5A—C6A	122.1 (2)
C4B—C5B—H5	118.8	C4A—C5A—H5A	118.9
C6B—C5B—H5	118.8	C6A—C5A—H5A	118.9
C5B—C6B—C7B	120.00 (19)	C5A—C6A—C7A	119.76 (19)
C5B—C6B—C1B	118.7 (2)	C5A—C6A—C1A	118.9 (2)
C7B—C6B—C1B	121.29 (18)	C7A—C6A—C1A	121.30 (19)
C8B—C7B—C6B	115.83 (19)	C8A—C7A—C6A	115.99 (19)
C8B—C7B—C12B	124.0 (2)	C8A—C7A—C12A	123.4 (2)
C6B—C7B—C12B	120.1 (2)	C6A—C7A—C12A	120.5 (2)
C7B—C8B—C9B	123.7 (2)	C7A—C8A—C9A	123.6 (2)
C7B—C8B—N8B	121.4 (2)	C7A—C8A—N8A	121.6 (2)
C9B—C8B—N8B	114.8 (2)	C9A—C8A—N8A	114.7 (2)
O81B—N8B—O82	80.8 (4)	O84—N8A—O83A	70.0 (14)
O81B—N8B—O82B	125.3 (4)	O84—N8A—O84A	79.0 (12)
O82—N8B—O82B	70.5 (4)	O83A—N8A—O84A	122.3 (3)
O81B—N8B—O81	72.2 (4)	O84—N8A—O83	130.3 (11)
O82—N8B—O81	122.9 (3)	O83A—N8A—O83	87.0 (8)
O82B—N8B—O81	85.6 (4)	O84A—N8A—O83	77.5 (7)
O81B—N8B—C8B	117.7 (3)	O84—N8A—C8A	120.0 (9)
O82—N8B—C8B	120.3 (3)	O83A—N8A—C8A	117.8 (2)
O82B—N8B—C8B	116.9 (3)	O84A—N8A—C8A	119.7 (2)
O81—N8B—C8B	116.8 (3)	O83—N8A—C8A	109.7 (6)
C10B—C9B—C8B	122.2 (2)	C10A—C9A—C8A	122.1 (2)
C10B—C9B—H9	118.9	C10A—C9A—H9A	119.0
C8B—C9B—H9	118.9	C8A—C9A—H9A	119.0
C9B—C10B—C1B	117.35 (18)	C9A—C10A—C1A	117.62 (18)
C9B—C10B—C13B	116.21 (18)	C9A—C10A—C13A	116.26 (18)
C1B—C10B—C13B	126.32 (18)	C1A—C10A—C13A	126.00 (18)
C3B—C11B—H11A	109.5	C3A—C11A—H11D	109.5
C3B—C11B—H11B	109.5	C3A—C11A—H11E	109.5
H11A—C11B—H11B	109.5	H11D—C11A—H11E	109.5
C3B—C11B—H11C	109.5	C3A—C11A—H11F	109.5
H11A—C11B—H11C	109.5	H11D—C11A—H11F	109.5
H11B—C11B—H11C	109.5	H11E—C11A—H11F	109.5
C7B—C12B—H12A	109.5	C7A—C12A—H12D	109.5
C7B—C12B—H12B	109.5	C7A—C12A—H12E	109.5
H12A—C12B—H12B	109.5	H12D—C12A—H12E	109.5
C7B—C12B—H12C	109.5	C7A—C12A—H12F	109.5
H12A—C12B—H12C	109.5	H12D—C12A—H12F	109.5
H12B—C12B—H12C	109.5	H12E—C12A—H12F	109.5
C15B—C13B—C14B	110.56 (19)	C14A—C13A—C10A	111.4 (2)
C15B—C13B—C10B	111.11 (18)	C14A—C13A—C15A	111.1 (2)
C14B—C13B—C10B	111.19 (19)	C10A—C13A—C15A	110.39 (18)
C15B—C13B—H13	107.9	C14A—C13A—H13A	107.9
C14B—C13B—H13	107.9	C10A—C13A—H13A	107.9
C10B—C13B—H13	107.9	C15A—C13A—H13A	107.9
C13B—C14B—H14A	109.5	C13A—C14A—H14D	109.5
C13B—C14B—H14B	109.5	C13A—C14A—H14E	109.5
H14A—C14B—H14B	109.5	H14D—C14A—H14E	109.5
C13B—C14B—H14C	109.5	C13A—C14A—H14F	109.5
H14A—C14B—H14C	109.5	H14D—C14A—H14F	109.5
H14B—C14B—H14C	109.5	H14E—C14A—H14F	109.5
C13B—C15B—H15A	109.5	C13A—C15A—H15D	109.5
C13B—C15B—H15B	109.5	C13A—C15A—H15E	109.5
H15A—C15B—H15B	109.5	H15D—C15A—H15E	109.5
C13B—C15B—H15C	109.5	C13A—C15A—H15F	109.5
H15A—C15B—H15C	109.5	H15D—C15A—H15F	109.5
H15B—C15B—H15C	109.5	H15E—C15A—H15F	109.5

### Hydrogen-bond geometry (Å, º)

**Table:**

*D*—H···*A*	*D*—H	H···*A*	*D*···*A*	*D*—H···*A*
C11*B*—H11*C*···O82^i^	0.98	2.42	3.243 (6)	142
C11*A*—H11*F*···O84*A*^ii^	0.98	2.43	3.240 (5)	139

Symmetry codes: (i) −*x*+1, −*y*, −*z*+1; (ii) −*x*+1, −*y*+1, −*z*.
